# Datopotamab deruxtecan in solid tumors: Recent research progress and clinical applications

**DOI:** 10.3389/fonc.2026.1870658

**Published:** 2026-07-07

**Authors:** Ying Cao, Li-qin Mo, Ya-juan Liu, Zhen Zhang

**Affiliations:** Department of Medical Oncology (III), Hangzhou Cancer Hospital, Hangzhou, China

**Keywords:** antibody-drug conjugate, clinical trial, datopotamab deruxtecan, safety, solid tumors

## Abstract

Following the approval of the first antibody-drug conjugate (ADC) for solid tumors, the therapeutic landscape in cancer has been revolutionized. Datopotamab deruxtecan (Dato-DXd) is a TROP2-targeting ADC which is composed of a humanized monoclonal antibody and a potent topoisomerase I inhibitor via a tetrapeptide-based cleavable linker. Based on data from multiple clinical trials, Dato-DXd has demonstrated compelling efficacy and a manageable safety profile. Herein, we provide an overview of recent published clinical trial data on Dato-DXd across various solid tumors. In addition, we propose future research directions to address critical issues in the clinical application of Dato-DXd, including optimal sequencing strategies with other TROP2-targeting ADCs, approaches to overcome drug resistance, and management of treatment-related adverse events.

## 1 Introduction

Over the past century, traditional chemotherapy has served as the cornerstone of cancer treatment. Although the advent of immunotherapy and targeted therapy has improved outcomes in certain malignancies, significant challenges remain, particularly for refractory cancers. As early as the beginning of the 20th century, Paul Ehrlich, the pioneer of chemotherapy, first proposed the concept of “Magic Bullet,” which laid the theoretical foundation for antibody-drug conjugates (ADCs) (1). In 1967, the ADC concept was formally proposed. Following the development of hybridoma technology, which enabled the production of monoclonal antibodies, ADC research began to accelerate significantly. In 2000, the U.S. Food and Drug Administration (FDA) granted accelerated approval to Mylotarg^®^ (gemtuzumab ozogamicin), marking the first approved ADC for the treatment of acute myeloid leukemia in adults (2). This milestone signified the beginning of the ADC era in targeted cancer therapy. ADCs are novel targeted anticancer drugs that combine the specificity of monoclonal antibodies with the potent cytotoxicity of small-molecule cytotoxic agents (3, 4). They consist of three components: a targeting antibody that recognizes tumor-specific antigens, a cytotoxic payload that a small-molecule cytotoxic drug is responsible for killing tumor cells, and a cleavable linker that connects the antibody to the cytotoxic payload and controls drug release. ADCs bind with high affinity to tumor-specific antigens, undergo endocytosis, deliver the payload to lysosomes, and induce cancer cell death. To date, ADC technology has progressed through three generations of development (5). In comparison to early ADCs, the new generation ADCs can more precisely target and deliver payloads to tumor cells, thereby exerting anti-tumor effects while significantly reducing toxicity to normal tissues. Moreover, a bystander effect can also eliminate neighboring tumor cells that do not express the target antigen, facilitated by moderate linker stability or high payload membrane permeability (5, 6). Currently, ADCs have emerged as one of the most prominent research directions in the field of precision oncology.

Trophoblast cell surface antigen 2 (TROP2) is a type I transmembrane glycoprotein, which was initially discovered in human trophoblast cells (7). Encoded by the *TACSTD2* gene located at chromosome 1p32.1, TROP2 is not classified as an oncogene (8). Previous studies have proven that TROP2 participates in cell growth, transformation and proliferation, and is closely associated with tumorigenesis and cancer progression (9–11). TROP2 also mediates multiple signaling pathways, including MAPK, JAK/STAT3, WNT/β-catenin, PTEN/PI3KCA/Akt, ErbB, TGFβ and NF-kB (9, 10, 12). TROP2 has been detected overexpressed across a broad spectrum of solid tumors, including lung, breast, esophageal, gastric, hepatic, pancreatic, renal, prostate, colorectal and ovarian cancers (8, 9, 11–15), with expression rates ranging from 18% to 99.3% (9, 12, 16, 17). In contrast, its expression in normal tissues is low (11). This distinct expression pattern renders TROP2 an ideal therapeutic target for solid tumors. Previous investigations have indicated that high TROP2 expression is associated with poor prognosis (12, 17, 18). These findings further support the necessity and therapeutic value of targeting TROP2 in cancer treatment.

To date, the application of TROP2-targeting ADCs in solid tumors has achieved landmark success. As of March 2026, three TROP2-targeting ADCs have been approved for clinical application: sacituzumab govitecan (SG), datopotamab deruxtecan and sacituzumab tirumotecan (sac-TMT/SKB264). Concurrently, numerous other TROP2-targeting ADCs are under investigation in preclinical and clinical settings. Moreover, the development of bispecific ADCs targeting TROP2—including VBC103 (19), AVZO-103 (20), and JSKN016 (21),—as well as combination therapy strategies, are also under exploration.

## 2 Overview of Dato-DXd

Datopotamab deruxtecan (DS-1062a/Dato-DXd; brand name: Datroway ^®^) is a TROP2 ADC composed of a humanized monoclonal antibody (datopotamab) linked via a tetrapeptide-based cleavable linker to a potent topoisomerase I inhibitor (DXd) (22). Dato-DXd exerts anti-tumor activity though both direct payload delivery and a bystander effect. Okajima et al. first discovered that Dato-DXd exhibited antitumor activity both *in vitro* and *in vivo* by inducing DNA damage and apoptosis in various TROP2-high expression tumor models, rather than in TROP2-low expression models (22). They subsequently launched the first-in-human clinical trial, the phase I TROPION-PanTumor01 study, to further verify the anti-tumor activity of Dato-DXd in solid tumors. To date, Dato-DXd has been approved to treat adult patients with hormone receptor-positive, HER2-negative (HR+/HER2-) breast cancer who have previously received endocrine-based therapy and chemotherapy for unresectable or metastatic disease (first approved in December 2024 by Japan) and adult patients with locally advanced or metastatic epidermal growth factor receptor (*EGFR*) -mutant non-small cell lung cancer (NSCLC) who have experienced disease progression despite prior EGFR-targeted therapy and platinum-based chemotherapy (approved by FDA in June 2025). In addition, Dato-DXd has exhibited robust antitumor activity across multiple solid tumor types. A summary of ongoing global clinical trials of Dato-DXd is provided in **Table 1**.

**Table 1:** Recruiting clinical trials on Dato-DXd.

Number	NCT number	Drug	Histology	Phase	Primary endpoint	Completion date
Breast cancer						
1	NCT06533826	Dato-DXd	BC	II	ORR	8/1/2038
2	NCT06103864	Dato-DXd	TNBC	III	PFS	9/30/2030
3	NCT06954480	Dato-DXd	TNBC	II	PFS	2/1/2030
4	NCT07069595	Dato-DXd	TNBC	II	The proportion of TNBC with MRD	11/1/2032
5	NCT06508216	Dato-DXd	TNBC	I/II	Part 1: RP2D; Part 2: ORR	12/17/2027
6	NCT05866432	Dato-DXd	TNBC with Brain Metastases	II	Intracranial ORR	5/23/2026
7	NCT06176261	Dato-DXd	HER2- BC with Brain Metastases	II	ORR	1/1/2029
8	NCT01042379	Dato-DXd	BC, Angiosarcoma	II	pCR Rate	12/1/2031
9	NCT07205822	Dato-DXd	HR+/HER2 IHC 0 BC	III	PFS	2/2/2028
NSCLC						
1	NCT06350097	Dato-DXd	EGFR-Mutant NSCLC	III	PFS	5/25/2032
2	NCT06417814	Dato-DXd	EGFR-Mutant NSCLC	III	PFS	9/27/2028
3	NCT06822543	Dato-DXd	NSCLC with Brain Metastases	II	Intracranial ORR	11/1/2028
4	NCT06357533	Dato-DXd	AGA-negative NSCLC	III	PFS	12/28/2029
5	NCT07291037	Dato-DXd	AGA-negative NSCLC	III	PFS, OS	1/29/2029
6	NCT05215340	Dato-DXd	AGA-negative NSCLC	III	PFS, OS	4/30/2028
7	NCT07535437	Dato-DXd	EGFR-Mutant NSCLC	I/II	Phase 1: DLTs, MTD; Phase 2: ORR	4/1/2028
8	NCT07098338	Dato-DXd	NSCLC	II	AE, ORR	4/6/2029
9	NCT05061550	Dato-DXd	NSCLC	II	pCR Rate, AE	5/28/2030
Other solid tumors						
1	NCT07129993	Dato-DXd	Urothelial Cancer, Bladder Cancer	II/III	Phase 2: ORR; Phase 3: PFS, OS	1/22/2030
2	NCT05489211	Dato-DXd	Solid Tumors	II	ORR, AEs, PFS	10/1/2027
3	NCT05417594	Dato-DXd	Solid Tumors	I/II	AEs, DLTs	8/11/2027
4	NCT06244485	Dato-DXd	Solid Tumors	I	Phase 1: AEs, DLTs; Phase 2: ORR	11/1/2028

Date collected until April 25, 2026. Date were obtained from clinicaltrials.gov. BC, breast cancer; TNBC, triple negative breast cancer; ER, estrogen receptor; HR, hormone receptor; HER2, human epidermal growth factor receptor 2; IHC, immunohistochemistry; NSCLC, Non-Small Cell Lung Cancer; AGA, Actionable Genomic Alterations; EGFR, Epidermal Growth Factor Receptor; pCR, pathologic complete response; ORR, overall response rate; PFS, progression-free survival; OS, overall survival; MRD, molecular residual disease; DLT, dose limiting toxicity; MTD, maximum tolerated dose; AEs, adverse events; RDE, Recommended Dose for Expansion; RP2D, recommended phase 2 dose.

This review provides a comprehensive overview of Dato-DXd across different types of solid tumors, including current research progress, clinical efficacy, and adverse reactions, in order to provide a reference for the subsequent development of Dato-DXd and patients selection for those who may derive therapeutic benefit.

## 3 Dato-DXd in NSCLC

### 3.1 Second-line and subsequent therapy

The phase I TROPION-PanTumor01 study comprised a dose-escalation phase to determine the maximum tolerated dose (MTD) and recommended dose for expansion (RDE), followed by a dose-expansion phase on the purpose of evaluating safety and tolerability in patients with heavily pretreated advanced or metastatic solid tumors, including TNBC, HR+/HER2- breast cancer, and NSCLC with or without actionable genomic alterations (AGAs) (23, 24). In the dose-escalation phase, patients received Dato-DXd at doses ranging from 0.27 to 10 mg/kg once every 3 weeks until the occurrence of unacceptable toxicity, progressive disease, or withdrawal of consent (23). Ultimately, the RDE of Dato-DXd was confirmed as 4, 6, or 8 mg/kg for the dose-expansion phase. At the data cutoff, 180 patients with advanced NSCLC had been enrolled. The confirmed objective response rate (ORR) was 22.0% for Dato-DXd 4 mg/kg, 26.0% for Dato-DXd 6 mg/kg, and 23.8% for Dato-DXd 8 mg/kg (**Table 2**). The median progression-free survival (PFS) was 6.9 months for Dato-DXd 6 mg/kg, with a corresponding median overall survival (OS) of 11.4 months. Notably, TROP2 expression was not correlated with therapeutic response. The incidence of grade ≥3 treatment-emergent adverse events (TEAEs) was 14% at 4 mg/kg, 26.0% at 6 mg/kg, and 36.3% at 8 mg/kg. Discontinuation owing to TEAEs occurred in 16.0%, 14.0%, and 23.8% of patients receiving Dato-DXd 4, 6, and 8 mg/kg, respectively. Dose reduction owing to TEAEs occurred in 2.0%, 10.0%, and 27.5% of patients at Dato-DXd 4, 6, and 8 mg/kg, respectively. The most common TEAEs were nausea, stomatitis, and alopecia; the most common grade ≥3 TEAEs were pneumonia, anemia, and decreased lymphocyte count. Of note, 13 patients (26.0%) experienced any-grade ocular surface toxicities (OSTs). Three of them were grade 3 OSTs, including one keratitis and two ulcerative keratitis in Dato-DXd 6 and 8 mg/kg. Interstitial lung disease (ILD) adjudicated as drug-related was reported in five patients (10.0%), three patients (6.0%), and 11 patients (13.8%) receiving Dato-DXd 4, 6, and 8 mg/kg, respectively. Grade ≥3 ILD occurred in six patients, three of whom experienced grade 5 events in the Dato-DXd 8 mg/kg group (23). Therefore, 6 mg/kg administered once every 3 weeks was selected for further development. ,

**Table 2:** Clinical efficacy of Datopotamab deruxtecan in NSCLC.

Trial identifier	Phase	Enrollment	Population	Treatment(s)	Efficacy
TROPION-PanTumor01	1	148	Pretreated advanced NSCLC	Dato-DXd 4/6/8 mg/kg	ORR: • 4 mg/kg: 22.0% • 6 mg/kg: 26.0% • 8 mg/kg: 23.8%
TROPION-Lung01	3	604	Pretreated advanced NSCLC	Dato-DXd vs. docetaxel	Median PFS: 4.4 vs. 3.7 months (HR = 0.75); Median OS: 12.9 vs. 11.8 months (P = 0.530).
TROPION-Lung05	2	137	Advanced NSCLC with AGAs	Dato-DXd	ORR: 35.8% (95% CI, 27.8-44.4)
ICARUS-LUNG01	2	100	Advanced NSCLC	Dato-DXd	ORR: 26% (95% CI, 17.7-35.7)
ORCHARD	2	69	EGFR-mutant advanced NSCLC	Dato-DXd plus osimertinib (4 vs. 6 mg/kg)	ORR: 43% vs. 36%
TROPION-Lung04	1b	Cohort 2: 15; Cohort 4: 37; Cohort 5: 40.	Advanced NSCLC without AGAs	• Cohort 2: Dato-DXd + durvalumab • Cohort 4: Dato-DXd + durvalumab + carboplatin • Cohort 5: Dato-DXd + rilvegostomig	Cohort 2: ORR 53.3% (95% CI, 26.6-78.7) Cohort 4: ORR 56.8% (95% CI, 39.5-72.9) Cohort 5: ORR 57.5% (95% CI, 40.9-73.0)
TROPION-Lung02	1b	142	Advanced NSCLC without AGAs	Dato-DXd + pembrolizumab vs. Dato-DXd + pembrolizumab + platinum chemotherapy	In the treatment-naïve population: ORR: 54.8% vs. 55.6%
NeoCOAST-2	2	202	Stage IIA-IIIB NSCLC without sensitizing EGFR mutations or ALK translocations	• Arm 1: Durvalumab + platinum-doublet chemotherapy + oleclumab • Arm 2: Durvalumab + platinum-doublet chemotherapy + monalizumab • Arm 4: durvalumab + single-agent platinum chemotherapy + Dato-DXd	pCR rates: 20.3% vs. 25.5% vs. 35.2%

Dato-DXd, Datopotamab deruxtecan**;** NSCLC, Non-Small Cell Lung Cancer; AGA, Actionable Genomic Alterations; EGFR, Epidermal Growth Factor Receptor; *ALK*, Anaplastic Lymphoma Kinase; pCR, pathologic complete response; ORR, overall response rate; PFS, progression-free survival; OS, overall survival; CI, confidence interval; HR, hazard ratio.

The TROPION-Lung01 study was the first phase III clinical trial in the field of lung cancer to report positive results for PFS, aiming to compare the efficacy and safety of Dato-DXd versus docetaxel in patients with pretreated advanced or metastatic NSCLC, regardless of the presence of AGAs (25). A total of 604 patients were randomly assigned (1:1) to received either Dato-DXd 6 mg/kg or docetaxel 75 mg/m^2^ once every 3 weeks. Patients receiving Dato-DXd demonstrated significantly improved median PFS compared with docetaxel, 4.4 months versus 3.7 months, with a hazard ratio (HR) of 0.75 [95% confidence interval (CI), 0.62 to 0.91; *P* = 0.004]. OS showed a numerical benefit but did not reach statistical significance (12.9 months versus 11.8 months; *P* = 0.530). In subgroup analyses, superior clinical benefits were found in patients with non-squamous NSCLC, in whom the median PFS was 5.5 versus 3.6 months (HR = 0.63; 95% CI, 0.51 to 0.79) and the median OS was 14.6 versus 12.3 months (HR = 0.84; 95% CI, 0.68 to 1.05); however, in patients with squamous NSCLC, the median PFS was 2.8 versus 3.9 months (HR = 1.41; 95% CI, 0.95 to 2.08) and the median OS was 7.6 versus 9.4 months (HR = 1.32; 95% CI, 0.91 to 1.92) with Dato-DXd and docetaxel, respectively. The poorer efficacy identified in patients with squamous histology might have also negatively affected another primary endpoint of OS in the overall population. Furthermore, in patients with non-squamous tumors harboring AGAs, the PFS benefit was further expanded (5.7 versus 2.6 months; HR = 0.35; 95% CI, 0.21 to 0.60). These subgroup analyses helped inform the identification of the population most likely to benefit in subsequent studies. Regarding safety, the most frequently occurring any-grade treatment-related adverse events (TRAEs) with Dato-DXd were stomatitis (47.5%), nausea (34.0%) and alopecia (32.0%), which were more frequent than in the docetaxel group. The incidence of grade ≥3 TRAEs was 25.6%, including stomatitis (6.7%), anemia (4.0%), and adjudicated ILD/pneumonitis (3.7%). It was noteworthy that there was 8.8% of patients (n=26) receiving Dato-DXd experienced any-grade adjudicated drug-related ILD/pneumonitis compared with 4.1% of patients in the docetaxel group. Additionally, the incidence of oral mucositis/stomatitis was 55.2%, with 10.4% of cases resulting in dose reductions. The most common OSTs were increased lacrimation (7.7%) and dry eye (7.1%), both of which were mild or moderate in severity. The incidence of keratitis was 4.0%, with 1.3% classified as grade ≥3. Among the 127 patients with prior brain metastases (BM) enrolled, the median intracranial (IC) PFS was 5.1 months for Dato-DXd versus 4.4 months for docetaxel (HR = 0.65; 95% CI, 0.39 to 1.09). A *post-hoc* analysis based on the TROPION-Lung01 trial revealed that among patients with untreated BM or progression after prior radiotherapy (n = 68), the IC ORR was 15.8% for Dato-DXd versus 0% for docetaxel (26). Dato-DXd also achieved favorable IC PFS compared with docetaxel, 5.0 months versus 3.0 months (HR = 0.48; 95% CI, 0.23 to 0.98). All disease remission occurred in patients with non-squamous histological type. The improved IC PFS was consistent regardless of EGFR mutation status. Furthermore, among patients with measurable IC disease (n = 27), the IC ORR benefit was further expanded, 38% for Dato-DXd versus 0% for docetaxel, with corresponding DCR of 88% versus 36%, respectively. Thus, Dato-DXd may serve as a potentially effective therapeutic choice for patients with non-squamous NSCLC, particularly for those with brain metastases, regardless of EGFR mutation status.

The TROPION-Lung05 trial was a phase II clinical trial that facilitated the approval of Dato-DXd in patients with EGFR-mutant NSCLC (27). It aimed to evaluate the efficacy and safety of Dato-DXd in patients with advanced or metastatic NSCLC harboring AGAs who had progressed after at least one line of targeted therapy and platinum-based chemotherapy. The confirmed ORR among all participants was 35.8% (95% CI, 27.8% to 44.4%). Among the 137 patients enrolled, 56.9% carried EGFR mutations, with an ORR of 43.6%. In patients who received osimertinib or harbored T790M mutations, the ORR reached 49.1%. Whereas, the ORR was 23.5% (95% CI, 10.7% to 41.2%) in patients with anaplastic lymphoma kinase (ALK) rearrangements. *Post-hoc* analysis of intracranial efficacy based on TROPION-Lung05 demonstrated that the IC ORR and DCR in 18 patients with measurable IC target lesions were 22% (95% CI, 6% to 48%) and 72% (95% CI, 47% to 90%), respectively, with a median time to IC response of 1.5 months and a median duration of IC response of 5.5 months (28). These results were consistent in patients with and without baseline brain metastases. The intracranial efficacy of Dato-DXd demonstrated consistency between the TROPION-Lung01 and TROPION-Lung05 trials, further supporting its intracranial activity.

In a pooled analysis of Dato-DXd in 117 patients with pretreated *EGFR*-mutant NSCLC from both the TROPION-Lung05 and TROPION-Lung01 trials, the confirmed ORR was 43% (95% CI, 34% to 52%), including 5 complete responses (CRs, 4%) (29). The median PFS and OS were 5.8 months (95% CI, 5.4 to 8.2) and 15.6 months (95% CI, 13.1 to 19.0), respectively. Antitumor activity was observed across a range of *EGFR* mutation subtypes. This further supported the application of Dato-DXd in patients with EGFR mutations.

The ICARUS-LUNG01 study was a phase II study aimed at assessing the efficacy and safety of Dato-DXd in patients with advanced NSCLC who had progressed on 1–3 previous standard therapies. As of April 18, 2024, the investigator-assessed confirmed ORR was 26% (95% CI, 17.4% to 34.6%), with a median PFS of 3.6 months. Patients with non-squamous NSCLC had a better ORR and median PFS of 30.5% and 4.8 months, respectively. The efficacy and safety profile data were consistent with those observed in the TROPION-Lung01 study. No correlation was detected between efficacy or resistance and genomic driver alterations.

The ORCHARD trial was a phase II study aiming to characterize resistance mechanisms and evaluate novel treatment combinations in patients with EGFR-mutated advanced NSCLC who had progressed on first-line osimertinib (30). A total of 69 patients were randomized at a ratio of 1:1 to receive oral osimertinib (80 mg once daily) plus intravenous Dato-DXd (4 mg/kg or 6 mg/kg every 3 weeks). In the 4 mg/kg and 6 mg/kg group, the confirmed ORR was 43% versus 36%, respectively; median PFS and OS were 9.5 and 19.8 months versus 11.7 and 26.2 months, respectively. The safety profiles were consistent with those from previous studies. Notably, Dato-DXd at 6 mg/kg combined with osimertinib exhibited superior survival benefit in the setting of osimertinib resistance. So, the combination of Dato-DXd and osimertinib constitutes a viable therapeutic strategy for overcoming osimertinib resistance. This positive outcome also supports Dato-DXd combination with osimertinib as first-line therapy for EGFR-mutant advanced NSCLC, offering the possibility of prolonged clinical response relative to osimertinib alone. However, this hypothesis requires validation through large-scale prospective randomized clinical trials.

### 3.2 First-line therapy

TROPION-Lung04 was a phase 1b, open-label, dose-escalation, and expansion study in patients with advanced or metastatic NSCLC without AGAs (31). In cohort 5, treatment-naïve patients received Dato-DXd plus rilvegostomig [a bispecific antibody targeting programmed cell death 1 (PD-1) and TIGIT] every 3 weeks (31). The ORR and DCR were 57.5% and 95.0%, respectively. Encouraging antitumor activity was observed across various levels of programmed cell death ligand 1 (PD-L1) expression and histologic types. Toxicities were manageable, and no new safety signals were identified. Based on these findings, the phase III TROPION-Lung10 study is ongoing to assess the efficacy of first-line Dato-DXd plus rilvegostomig versus pembrolizumab in patients with advanced or metastatic NSCLC with high PD-L1 expression and without AGAs (32). In cohort 2 (C2) and cohort 4 (C4), enrolled patients were treatment-naïve or had received one prior line of chemotherapy for advanced or metastatic NSCLC (33). Patients in C2 and C4 received Dato-DXd plus durvalumab every 3 weeks, and patients in C4 also received carboplatin (4 cycles) every 3 weeks. At data cut-off (May 23, 2024), 52 patients received 1L treatment with Dato-DXd + durvalumab ± carboplatin. Confirmed ORR and DCR was 53.3% and 86.7% for C2, and 56.8% and 89.2% for C4, respectively. Additionally, the median PFS was 7.3 months (95% CI, 2.0 to 29.5) in C2 and 8.7 months (95% CI, 5.6 to 10.1) in C4, respectively. Responses were observed across all evaluated PD-L1 levels and in both squamous and non-squamous subgroups. In C2, the confirmed ORRs were 50.0% and 54.5% in the squamous and non-squamous subgroups, respectively. In C4, the confirmed ORRs were 47.4% and 66.7% in the squamous and non-squamous subgroups, respectively. Unlike prior single-agent studies of Dato-DXd, combination therapy with Dato-DXd has demonstrated clinical efficacy in advanced squamous NSCLC. This represents a novel therapeutic approach for advanced squamous NSCLC. Additionally, Dato-DXd combination regimens provided benefit across PD-L1 expression subgroups, with similar ORRs observed in cohorts 2 and 4, potentially enabling advanced NSCLC patients without AGAs to avoid chemotherapy. Nevertheless, these results are limited by small sample sizes and necessitate confirmation through large-scale prospective clinical trials.

TROPION-Lung02 was also a phase 1b, open-label, dose-escalation, and expansion study (six-cohort design) in patients with advanced or metastatic NSCLC without AGAs (34). Eligible patients in cohorts 1–2 were assigned to receive Dato-DXd (4 or 6 mg/kg) plus pembrolizumab 200 mg (doublet therapy) every 3 weeks. Eligible patients in cohorts 3–6 received Dato-DXd (4 or 6 mg/kg) plus pembrolizumab 200 mg combined with platinum chemotherapy (triplet therapy) every 3 weeks. Ultimately, 70 and 72 patients received doublet and triplet therapy, respectively. Baseline demographics were similar between the two groups, except for the proportion of treatment-naïve patients. There were 42 (60%) versus 54 (75%) treatment-naïve patients in the doublet and triplet therapy groups, respectively. In the treatment-naïve population (n=96), the confirmed ORR was 54.8% versus 55.6% for doublet and triplet therapy, respectively. The median duration of response (DOR) and PFS were 20.1 and 11.2 months for doublet therapy, compared with 13.7 and 6.8 months for triplet therapy, respectively. The median OS was not mature for doublet therapy and was 17.4 months for triplet therapy. Tumor responses were observed regardless of PD-L1 expression for both regimens. TROP2 normalized membrane ratio (NMR) biomarker analyses indicated a trend between improved survival outcomes and positive biomarker status. The incidence of grade ≥3 TRAEs was 37.1% for doublet therapy and 59.7% for triplet therapy, which were consistent with those observed for each agent individually. The incidence of grade ≥3 oral mucositis/stomatitis, ocular surface events and adjudicated drug-related ILD/pneumonitis was 7% versus 4%, 1% versus 3%, and 3% versus 1% for doublet and triplet therapy, respectively. No treatment-related deaths were reported. Therefore, Dato-DXd plus pembrolizumab represents a promising first-line option with acceptable safety profiles and robust antitumor activity for untreated advanced NSCLC without AGAs, particularly in patients with low PD-L1 expression levels. This represents a paradigm shift that could fundamentally challenge platinum-based chemotherapy’s position as the standard first-line treatment in advanced NSCLC without AGAs.

### 3.3 Perioperative therapy

The phase II NeoCOAST-2 trial was designed to identify the superior neoadjuvant treatment regimen in patients with untreated, resectable stage IIA-IIIB NSCLC without sensitizing *EGFR* mutations or *ALK* translocations (35). Eligible candidates were assigned to receive neoadjuvant durvalumab plus platinum-doublet chemotherapy combined with either oleclumab (a CD73 inhibitor; Arm 1), monalizumab (a NKG2A inhibitor; Arm 2), or neoadjuvant durvalumab plus single-agent platinum chemotherapy combined with Dato-DXd (Arm 4). All patients subsequently underwent surgical resection. Thereafter, patients continued to receive adjuvant durvalumab with oleclumab or monalizumab (Arms 1 or 2) or durvalumab alone (Arm 4). The primary endpoints were pathological complete response (pCR) rate and safety. The pCR rates in the modified intention-to-treat (mITT) population were 20.3% (95% CI, 11.8% to 31.2%) in Arm 1, 25.7% (95% CI, 16.0% to 37.6%) in Arm 2, and 35.2% (95% CI, 22.7% to 49.4%) in Arm 4. In Arm 4, pCR rates were consistent across various levels of PD-L1 expression. The median percentage of partial response (PR) rates in mITT population were 41.9% in Arm 1, 50.0% in Arm 2, and 63.0% in Arm 4. Additionally, the median percentage of residual viable tumor (%RVT) was lowest in Arm 4 at 4.5%, compared with 20.0% in Arm 1 and 10.0% in Arm 2. During the neoadjuvant period, the overall incidence of grade ≥3 adverse events (AEs) was 36.5% in Arm 1, 42.3% in Arm 2, and 24.1% in Arm 4. Furthermore, grade ≥3 AEs possibly related to oleclumab, monalizumab, or Dato-DXd occurred in 14.9%, 16.9%, and 16.7% of patients in Arms 1, 2, and 4, respectively. The most common AEs in Arm 4 were anemia (44.4%), asthenia (42.6%), alopecia (31.5%), and thrombocytopenia (31.5%). Thus, neoadjuvant Dato-DXd plus durvalumab with single-agent platinum demonstrated a favorable pCR rate and a manageable safety profile in patients with resectable NSCLC. Moreover, the pCR rate observed in the Dato-DXd plus durvalumab with single-agent platinum regimen significantly exceeded the 17.2% pCR rate previously reported in the Phase III AEGEAN trial for the durvalumab plus platinum-doublet chemotherapy regimen (36). This finding may challenge the current standard neoadjuvant treatment paradigm; however, head-to-head prospective clinical trials are needed to validate this conclusion.

A series of Dato-DXd clinical trials are currently ongoing, focusing on NSCLC. For example, the TROPION-Lung15 trial aims to compare the efficacy and safety of Dato-DXd as monotherapy and in combination with osimertinib versus platinum-based chemotherapy in patients with advanced or metastatic NSCLC harboring *EGFR* mutations who have progressed after osimertinib (37). In addition, the exploration of Dato-DXd in combination with immunotherapy for metastatic NSCLC without AGAs is ongoing. These include the TROPION-Lung07 (38), TROPION-Lung08 (39), and TROPION-Lung10 trials (32) as first-line therapy, as well as the TROPION-Lung12 trial as adjuvant therapy (40). The results from these trials eagerly awaited, as they are expected to provide new treatment options for this patient population.

## 4 Dato-DXd in breast cancer

### 4.1 Second-line and subsequent therapy

In the breast cancer cohort of TROPION-PanTumor01, 41 patients with advanced HR+/HER2- breast cancer and 44 with advanced TNBC were recruited (24). The confirmed ORR of Dato-DXd were 26.8% and 31.8% in HR+/HER2- breast cancer and TNBC, respectively (**Table 3**). The corresponding median PFS by blinded independent central review (BICR) was 8.3 months in HR+/HER2- breast cancer and 4.4 months in TNBC. In the topoisomerase I-naïve TNBC subgroup, patients demonstrated higher ORR and longer median PFS. The incidence of grade ≥3 TEAEs was 22.0% in HR+/HER2- breast cancer and 25.0% in TNBC. Only one patient with HR+/HER2- breast cancer experienced grade 3 ILD. OSTs occurred in 41.5% and 36.4% of patients with HR+/HER2- breast cancer and TNBC, respectively. Grade ≥3 oral mucositis/stomatitis occurred in 9.8% and 11.4% of patients with HR+/HER2- breast cancer and TNBC, respectively. Neither grade ≥3 infusion-related reactions (IRRs) nor any grade 5 events were reported.

**Table 3:** Clinical efficacy of Datopotamab deruxtecan in breast cancer.

Trial identifier	Phase	Enrollment	Population	Treatment(s)	Efficacy
TROPION-PanTumor01	1	85	Advanced HR+/HER2- BC and TNBC	Dato-DXd	ORR: • HR+/HER2- BC: 26.8% (95% CI, 14.2-42.9) • TNBC: 31.8% (95% CI, 18.6-47.6)
TROPION-Breast01	3	732	Pretreated advanced HR+/HER2- BC	Dato-DXd vs. chemotherapy	Median PFS: 6.9 vs. 4.9 months (HR = 0.63) Median OS: 18.6 vs. 18.3 months (HR = 1.01)
TUXEDO-2	2	8	TNBC with brain metastases	Dato-DXd	Intracranial ORR: 37.5%
DATO-Base	2	10	HER2-negative BC with brain or leptomeningeal metastases	Dato-DXd	ORR: 30.0% Median intracranial PFS: 4.1 months (95% CI, 0.7 to not reached)
TROPION-Breast02	3	644	Untreated advanced TNBC and not suitable for immunotherapy	Dato-DXd vs. chemotherapy	Median PFS: 10.8 vs.5.6 months (HR = 0.57) Median OS: 23.7 vs. 18.7 months (HR = 0.79)
BEGONIA	1b/2	47	Untreated advanced TNBC	Dato-DXd + durvalumab	ORR: 79.0% (95% CI, 61-91)
I-SPY2.2	2	106	Stage II-III BC	Dato-DXd + durvalumab	pCR rate: 77.0%

Dato-DXd, Datopotamab deruxtecan**;** BC, breast cancer; TNBC, triple negative breast cancer; HR, hormone receptor; HER2, human epidermal growth factor receptor 2; pCR, pathologic complete response; ORR, overall response rate; PFS, progression-free survival; OS, overall survival; CI, confidence interval; HR, hazard ratio.

The TROPION-Breast01 study was a global, phase III, randomized trial designed to assess Dato-DXd versus investigator’s choice of chemotherapy (ICC) in patients with unresectable or metastatic HR+/HER2- breast cancer who had progressed on endocrine therapy and one to two lines of chemotherapy in the unresectable or metastatic setting (41). The primary endpoints were PFS and OS by BICR. Dato-DXd was administered at 6 mg/kg once every 3 weeks. As a result, there were 365 patients receiving Dato-DXd and 367 patients receiving ICC. Dato-DXd demonstrated more favorable PFS compared with ICC (6.9 months versus 4.9 months; HR = 0.63, *P* < 0.001) (41). The PFS benefit was consistent across all subgroups, regardless of the number of prior lines of therapy and endocrine therapy sensitivity. The ORR with Dato-DXd was 36.4%, superior to that with ICC (22.9%). Dato-DXd had less grade ≥3 TRAEs than ICC (20.8% versus 44.7%, respectively). The most frequently reported grade ≥3 TRAEs in the Dato-DXd group were stomatitis (6.4%), fatigue (1.7%), and nausea (1.4%). No fatal TRAEs were reported in the Dato-DXd group. The incidence of ILD/pneumonitis in the Dato-DXd group was 3.3%, predominantly grade 1 or 2. The positive outcomes of TROPION-Breast01 supported FDA approval of Dato-DXd for unresectable or metastatic HR+/HER2- breast cancer following progression on endocrine therapy and one to two lines of chemotherapy in the unresectable or metastatic setting. After a median follow-up of 22.8 months, the final OS analysis revealed that median OS was 18.6 months (95% CI, 17.3 to 20.1) with Dato-DXd compared with 18.3 months (95% CI, 17.3 to 20.5) with ICC (HR = 1.01; 95% CI, 0.83 to 1.22; *P* = 0.9445) (42). The OS rates at 6, 12, and 18 months were 93.3%, 74.2%, and 52.0% for Dato-DXd versus 87.9%, 71.1%, and 50.9% for ICC, respectively. Of note, the proportion of patients receiving subsequent therapy was 73.7% in the Dato-DXd group versus 78.7% in the ICC group. Moreover, 24.0% of patients in the ICC group received an ADC as subsequent therapy (22% for trastuzumab deruxtecan [T-DXd], 7% for SG, and 5% for both), which was substantially higher than the proportion in the Dato-DXd group (12.3%) and may have influenced the OS results. Furthermore, in the HER2 immunohistochemistry (IHC) 0 subgroup, Dato-DXd showed a trend toward improved OS, with median OS of 17.8 months versus 14.3 months (HR = 0.89; 95% CI, 0.64 to 1.24).

TUXEDO-2 was a phase II study designed to evaluate the efficacy and safety of Dato-DXd in patients with TNBC and newly diagnosed, untreated, or progressing BM, without indications for immediate local therapy (43). The primary endpoint was intracranial response rate. The trial was a two-stage design: if ≥2 responses were observed in the first stage, the trial would proceed to the second stage. Eight eligible patients were recruited in the first stage, three of whom had received prior ADCs (2 for SG and 1 for T-DXd). As a result, five patients were evaluable and three of them had intracranial response, with an intracranial response rate of 37.5%. Notably, all three responders were ADC-naïve. Thus, the first stage of TUXEDO-2 was successful, and the second stage is currently ongoing. This finding raises the clinical question of cross-resistance among ADCs: after failure of an ADC with a specific target or payload, whether another ADC targeting the same antigen or carrying a similar payload remains effective in later-line settings.

DATO-Base was another single-arm phase II study designed to explore the intracranial activity of Dato-DXd in patients with advanced HER2-negative breast cancer and active BMs and/or leptomeningeal disease (LMD) (44, 45). The study contained three cohorts and the Cohort C was for HER2-negative breast cancer with LMD, either newly diagnosed or progressive. A total of 10 patients were enrolled, who had received a median of 2.5 prior lines of cytotoxic treatment, including ADCs (45). Eight patients had previously experienced local therapy for central nervous system disease. According to Leptomeningeal Assessment in Neuro-Oncology criteria, the ORR was 30%, with a median IC PFS and OS of 4.1 months (95% CI, 0.7 to not reached) and 4.7 months (95% CI, 0.7 to not reached), respectively. Similar to studies in lung cancer, this demonstrates that Dato-DXd also has intracranial activity in breast cancer with LMD, making it a preferred option for patients with central nervous system disease. Furthermore, compared with ADC-pretreated patients, ADC-naïve patients (n=3) had numerically longer IC PFS (5.9 versus 1.4 months) and OS (13.3 versus 3.7 months). Thus, these findings hint at the possibility of cross-resistance in ADC re-challenge scenarios, albeit based on a limited sample size.

### 4.2 First-line therapy

The phase III TROPION-Breast02 trial was designed to evaluate the efficacy and safety of Dato-DXd versus investigator’s choice of chemotherapy in patients with previously untreated, locally recurrent inoperable or metastatic TNBC, who were not candidates for PD-1/PD-L1 inhibitor therapy (46). The results were first presented at the 2025 Annual Congress of the European Society for Medical Oncology (47). As of August 25, 2025, a total of 644 patients had been randomly assigned in a 1:1 ratio to receive either Dato-DXd or chemotherapy. Compared with chemotherapy, Dato-DXd significantly improved both PFS and OS (10.8 versus 5.6 months; HR = 0.57, *P* < 0.0001; 23.7 versus 18.7 months; HR = 0.79, *P* = 0.0291, respectively). Notably, Dato-DXd showed superior ORR and DOR compared with chemotherapy (62.5% versus 29.3% for ORR; 12.3 versus 7.1 months for DOR, respectively). The success of TROPION-Breast02 supports Dato-DXd as a novel standard first-line treatment for patients with locally recurrent, inoperable, or metastatic TNBC who are not suitable for immunotherapy.

The phase 1b/2 BEGONIA study investigated Dato-DXd plus the anti-PD-L1 antibody durvalumab as first-line treatment for advanced TNBC in Arm 7 (48, 49). At the data cutoff, the ORR was 79%, with all responding patients. The median PFS and DOR were 13.8 months and 15.5 months, respectively. The incidence of grade ≥3 TRAEs was 57%, and the safety profile was manageable. The most common AEs were nausea and stomatitis. Both ORR and PFS demonstrate numerical superiority for the Dato-DXd plus durvalumab combination compared with Dato-DXd monotherapy in TROPION-Breast02 and pembrolizumab plus chemotherapy in KEYNOTE-355 (the ORR was 53%, and median PFS was 9.7 months) (50), indicating potential synergism between Dato-DXd and immune checkpoint inhibitors. This combination may also offer an alternative to conventional chemotherapy regimens for patients with advanced TNBC, pending validation in adequately powered prospective trials.

The exploration of Dato-DXd in combination with immunotherapy for TNBC or HR+/HER2- breast cancer is ongoing, including the TROPION-Breast03 trial as adjuvant therapy (51), the TROPION-Breast04 trial as neoadjuvant therapy (52), and the TROPION-Breast05 trial as first-line therapy (53). Results from these trials are eagerly anticipated, with the hope of providing new treatment options for these patients.

### 4.3 Perioperative therapy

I-SPY2.2 was a signal-finding trial designed to screen highly effective regimens that predict a high probability of success in phase III trials. The I-SPY2.2 was a phase II neoadjuvant randomization trial for stage 2/3 breast cancer. The trial consisted of three sequential treatment blocks: Block A, involving initial randomization to different experimental agent(s); Block B, with a taxane-based regimen tailored to tumor subtype; and Block C, with doxorubicin-cyclophosphamide (54). Dato-DXd plus durvalumab (Block A) achieved a pCR rate of 54% in the immune-positive subtype. The overall pCR rate for the entire treatment strategy (Dato-DXd + durvalumab → paclitaxel + carboplatin + pembrolizumab → doxorubicin + cyclophosphamide + pembrolizumab) was 77% (54). Notably, 92% of patients in the immune-positive group avoided the doxorubicin-cyclophosphamide chemotherapy. Besides, neoadjuvant combination therapy resulted in pCR rates of 10% for HR+/HER2- breast cancer and 33% for TNBC (54). However, pCR rates with Dato-DXd alone were 6% for HR+/HER2- disease, 30% for TNBC, and 41% for TNBC with immune-DNA repair deficiency subtype (55). No new safety signals were identified. This also indicates that there is indeed a synergistic effect between Dato-DXd and durvalumab. Thus, Dato-DXd plus durvalumab represents an encouraging therapeutic combination for neoadjuvant therapy in breast cancer, particularly for the immune-positive subtype. Nevertheless, more research with larger sample sizes is required to validate these findings.

## 5 Dato-DXd in other solid tumors

Inspired by its remarkable success in breast and lung cancer, Dato-DXd has also been investigated in other solid tumors (**Table 1**), although most of these studies have focused on preclinical research. Several published preclinical studies have demonstrated that Dato-DXd exhibits promising antitumor activity against TROP2-overexpressing ovarian cancer, uterine serous carcinoma, poorly differentiated endometrial carcinoma, cervical carcinoma, as well as uterine and ovarian carcinosarcoma (56–60). Additionally, Dato-DXd plus AZD5305 (a selective inhibitor of Poly [ADP-ribose] polymerase 1 [PARP1]) demonstrated enhanced antitumor activity in a TROP2+/homologous recombination deficiency (HRD)-negative gastric cancer xenograft model and a TROP2+, PARP1 inhibitor-resistant ovarian cancer patient-derived xenograft (PDX) model (61). However, the antitumor activity of Dato-DXd in these tumors requires further confirmation from randomized clinical trials.

In the phase 2 TROPION-PanTumor03 study, 40 patients with recurrent unresectable advanced or metastatic endometrial carcinoma (EC) and 35 patients with recurrent unresectable advanced or metastatic high-grade serous ovarian, fallopian tube, or primary peritoneal carcinoma (OC) received Dato-DXd 6 mg/kg every 3 weeks; all had disease progression after ≥1 line of platinum-based chemotherapy (62). At the data cut-off (March 1, 2024), in the EC cohort, the confirmed ORR and DCR were 27.5% and 85.0%, respectively, with a median PFS of 6.3 months (95% CI, 2.8 to not yet reached); in the OC cohort, the corresponding ORR and DCR were 42.9% and 91.4%, respectively, with a median PFS of 5.8 months (95% CI, 4.1 to 7.1). The safety profile was manageable. TEAES were consistent with the known profile of Dato-DXd. Dato-DXd represents a promising therapeutic option for patients with advanced EC and OC after platinum-based chemotherapy failure. However, confirmation through large-scale randomized controlled trials is necessary before widespread clinical adoption.

In the phase 1 TROPION-PanTumor01 study, eighteen patients with unresectable locally advanced/metastatic urothelial cancer who had received ≥1 prior line of therapy were enrolled (63). Of these, 88.3% had received ≥3 prior regimens and 100% had received prior immunotherapy. After a median follow-up of 9.1 months, 33.3% patients were still receiving Dato-DXd 6 mg/kg every 3 weeks. The confirmed ORR and DCR were 27.8% and 77.8%, respectively. These findings demonstrate that Dato-DXd exhibited encouraging antitumor activity in patients with advanced urothelial cancer, including those with prior immunotherapy exposure. However, the generalizability of these findings is limited by the small sample size. While the safety profile was manageable, larger prospective randomized trials are needed to confirm efficacy in these settings.

## 6 Prospects and challenges

Currently, Dato-DXd has been approved to treat adult patients with HR+/HER2- breast cancer who have previously received endocrine-based therapy and chemotherapy for unresectable or metastatic disease and adult patients with locally advanced or metastatic *EGFR* -mutant NSCLC who have experienced disease progression despite prior *EGFR*-targeted therapy and platinum-based chemotherapy. In addition, Dato-DXd monotherapy and combination regimens with targeted and immune therapies have shown impressive clinical efficacy across NSCLC, HR+/HER2- breast cancer, and TNBC in both perioperative and first-line metastatic treatment settings. Furthermore, Dato-DXd has also exhibited favorable efficacy in the management of intracranial metastases in NSCLC and breast cancer. In preclinical TROP2-expressing intracranial tumor model, immunohistochemistry analysis confirmed that Dato-DXd was localized in intracranial tumor tissues, and Dato-DXd showed a significant survival benefit over control ADC (P = 0.0002) (64). However, the specific mechanism of Dato-DXd crossing the blood-brain barrier remains unclear. It is believed that in the near future, Dato-DXd will once again disrupt the current treatment landscape. Nevertheless, several critical issues require urgent investigation in the clinical application of Dato-DXd: (1) establishing optimal treatment sequencing with other TROP2-targeting ADCs; (2) exploring strategies to overcome drug resistance; and (3) implementing standardized management protocols for treatment-related toxicities.

First, with SG and sac-TMT approved for clinical use, the question arises: which agent should be selected as the initial therapy under the same indication? Furthermore, when resistance or disease progression occurs, except considering their specific toxicity profiles, can these agents be used sequentially? Moreover, the use of HER2-targeting ADCs has added additional complexity to these challenges. However, at present, there is no robust evidence to support optimal ADC treatment sequencing strategies. Several multicenter, retrospective studies of the sequential use of T-DXd and SG in patients with HER2-low metastatic breast cancer demonstrated that ADC1 had a longer median PFS or higher ORR than ADC2, regardless of ADC sequence order, or HR status (65–67). One retrospective study found that 54.5% of patients exhibited primary resistance to the second ADC (67). These findings indicate that the efficacy of a second ADC is reduced when using ADCs with similar payloads (topoisomerase I), even when targeting different antigens. Chen et al. conducted a retrospective study in patients with advanced HER2-positive or HER2-low breast cancer showing that payload switching between ADCs led to improved ORR (68). ADC2 with a similar payload to ADC1 yielded an ORR of 5.3%, whereas switching to a different payload increased the ORR to 22.6%. Another pilot study revealed that the drug payload, rather than the targeting antibody, contributed to acquired resistance in TROP2 and HER2-targeting ADCs (69). Rampa et al. also revealed that cross-resistance to sequential ADC was primarily driven by shared payload mechanisms rather than loss of target expression, as shown both *in vitro* and *in vivo* (70). After progression on a topoisomerase I inhibitor-based ADC, switching to ADCs carrying non-cross-resistant payload classes is recommended to restore antitumor activity. Nevertheless, a retrospective study with a limited sample size (n=68) reported conflicting conclusions, proposing that both antibody targets and payloads may potentially lead to cross-resistance (71). Therefore, real-world studies have confirmed the existence of cross-resistance among ADCs, particularly with ADCs sharing the same topoisomerase I inhibitor payload, though prospective studies are needed to validate these findings. In the TROPION-PanTumor01 trial, Dato-DXd demonstrated higher ORR and longer PFS in the topoisomerase I-naïve TNBC subgroup (24). This finding suggests potential cross-resistance between Dato-DXd and other ADCs utilizing topoisomerase I inhibitor payloads. At present, all three approved TROP2 ADCs employ topoisomerase I inhibitors as their payloads; consequently, sequential administration of Dato-DXd, SG, and sac-TMT may lead to cross-resistance, and this approach is not recommended. However, this requires prospective clinical trials to investigate the sequential administration of ADCs with target or payload switching. When disease progression or resistance occurs, enrollment in clinical trials is suggested, or switching to ADCs with different payloads. As reported in TROPION-Breast01, patients in the chemotherapy group had a remarkable higher proportion of receiving an ADC as subsequent therapy than those in the Dato-DXd group (24.0% versus 12.3%), which may confound survival outcome analyses (42). Therefore, the use of TROP2-targeting ADCs in subsequent treatment is an important confounding factor in ongoing clinical trials of new ADC development. The currently approved indications for the three TROP2 ADCs globally show significant overlap, yet their market availability varies across different regions. However, none of their pivotal registration trials were designed with direct head-to-head comparisons against other ADC therapies, resulting in no robust evidence to definitively rank their relative efficacy within shared indications. This presents a clinical challenge for treatment selection. In our opinion, when drug accessibility permits, clinicians should adopt individualized selection strategies tailored to each patient’s specific characteristics. First, the three TROP2 ADCs exhibit distinct adverse event profiles. The predominant ≥3 grade toxicity for Dato-DXd is stomatitis, with a lower incidence of chemotherapy-like adverse events; however, clinicians should also monitor for OSTs, drug-induced ILD, and infusion-related reactions. For SG, myelosuppression is the primary concern, with gastrointestinal events, especially diarrhea, requiring close attention (72, 73). Sac-TMT also requires vigilant monitoring of both myelosuppression and stomatitis (74, 75). Clinicians must also consider treatment goals. While sac-TMT demonstrates numerically superior ORR and PFS compared to SG and Dato-DXd across multiple tumor types, these findings are not derived from randomized head-to-head trials, and the limited sample sizes warrant cautious interpretation.

Drug resistance following Dato-DXd treatment is an inevitable challenge. The mechanisms of resistance to TROP2 ADCs have not yet been fully elucidated; however, several studies have revealed that downregulation or loss of TROP2 expression on the surface of tumor cells, resistance to the cytotoxic effect of ADCs, dysregulation of lysosomal acidification, loss of internalization pathways, and genomic alterations may all contribute to the development of resistance (11, 76–78). Therefore, overcoming resistance is of utmost importance. For example, novel ADCs are under development to enhance lysosomal stability, target additional oncogenic pathways, and innovate payload designs (78–80). Another approach is to investigate rational therapeutic partners to synergize with ADCs and counteract resistance, such as the TROPION-Lung02 and BEGONIA trials. In human lung cancer cells, DS-1103a (an anti-human SIRPα antibody) was observed to significantly enhance antibody-dependent cellular phagocytosis of Dato-DXd through blockade of the SIRPα-CD47 axis (81). The enhanced antitumor activity of combination therapy was further confirmed in syngeneic mouse models. Large-scale clinical trials are needed to validate the efficacy of these combination regimens.

Additional attention should be paid to screening for novel biomarkers to predict clinical efficacy and identify the patient population most likely to benefit. Although TROP2 has been reported to be widely overexpressed in solid tumors, the absence of standardized assays and validated cutoff values significantly hinders effective patient stratification in both research and clinical contexts. Previous studies have shown that TROP2 expression assessed by traditional immunohistochemical methods was not associated with tumor response, as exemplified in the TROPION-PanTumor01 study. Therefore, an urgent need exists for a highly reproducible, objective detection method to establish standardized criteria for TROP2 positivity. Garassino et al. proposed the hypothesis that a more precise and quantitative assessment of TROP2 expression on the cell membrane and in the cytoplasm might predict the therapeutic response to Dato-DXd (82). Based on this hypothesis, they developed the Quantitative Continuous Scoring (QCS) model. QCS is a fully supervised computational pathology algorithm trained on pathologist’s annotations to identify tumor areas and individual cellular compartments (membrane and cytoplasm) across whole-slide images. TROP2 NMR is measured using this QCS model, which quantifies the expression of TROP2 in the cell membrane relative to total TROP2 expression (membrane and cytoplasm) in tumor biopsy samples. Garassino and colleagues conducted an exploratory analysis of the phase III TROPION-Lung01 trial, and revealed that TROP2 NMR determined by QCS demonstrated potential as a predictive biomarker for Dato-DXd. In the non-squamous NSCLC without AGAs subgroup, TROP2 NMR+ patients had numerically longer median PFS and higher response rates than TROP2 NMR− patients in the Dato-DXd setting (PFS: 7.2 versus 4.0 months; ORR: 36.8% versus 22.5%), which was not observed in the docetaxel setting. Subsequently, in the phase Ib TROPION-Lung02 trial, TROP2 NMR biomarker analyses also exhibited an association between improved survival and positive biomarker status. The cutoff of TROP2 NMR was 0.56, which was less in at least 75% of the tumor cells in whole-slide image samples defined as TROP2 NMR+, otherwise defined as TROP NMR- (34). In the overall analyzed cohort, the confirmed ORR was 64.9% in TROP2 NMR+ patients versus 56.4% in TROP2 NMR- patients, with median PFS of 12.0 versus 8.1 months (HR = 0.62; 95% CI, 0.35 to 1.10), respectively. The median OS was 26.9 months versus 19.7 months in TROP2 NMR+ and TROP2 NMR− patients (HR = 0.59; 95% CI, 0.29 to 1.22), respectively. The results were consistent in patients receiving doublet or triplet therapy. These findings support TROP2 NMR as a potential predictive biomarker of Dato-DXd in NSCLC. However, whether this biomarker is applicable to other tumor types remains to be determined. Further confirmation in large-scale, prospective clinical trials is needed. However, the implementation of QCS is subject to certain practical constraints. As a proprietary computational pathology platform based on deep learning algorithms, QCS relies on specialized infrastructure that may not be available in all clinical settings. This platform dependence could potentially limit its widespread clinical adoption compared to traditional pathologist-led IHC scoring methods. Besides, novel biomarkers for combination therapy are under exploration. Moreover, HRD status may predict the efficacy of Dato-DXd in combination with a PARP inhibitor, but it showed heterogeneity across different cancer types. In preclinical models, Dato-DXd plus a PARP1 inhibitor demonstrated enhanced antitumor activity in a TROP2+/HRD-negative gastric cancer xenograft model; however, this enhanced antitumor activity appeared in NSCLC and TNBC cell regardless of HRD status (61). Another PDX model study in breast cancer discovered that Dato-DXd generated stronger antitumor effects in HRD-positive PDX models than HRD-negative PDX models, and the enhanced effect of the PARP inhibitor (olaparib) on Dato-DXd existed only in Dato-DXd-sensitive models, not in Dato-DXd-resistant models (83).

In addition to efficacy, clinicians should emphasize safety management. Evidence indicates that Dato-DXd had a lower incidence of grade ≥3 TRAEs than chemotherapy (20.8% versus 44.7%, respectively) and less hematological toxicity (41). A meta-analysis regarding the safety of Dato-DXd revealed that the incidence of grade ≥ 3 TRAEs was 28.7% (84). The most common any-grade TRAEs were stomatitis (55.1%), nausea (49.4%), alopecia (38.5%), fatigue (28.1%), and decreased appetite (19.1%); the most common grade ≥ 3 TRAEs were stomatitis (6.8%), anemia (2.9%), ILD (1.7%), fatigue (1.5%), and asthenia (1.3%) (84). ILD, an adverse event of special interest (AESI) associated with ADCs including Dato-DXd, occurred more frequently in patients with NSCLC than in patients with breast cancer, and this pattern may be attributed to underlying risk factors more prevalent in NSCLC populations, including smoking history and pre-existing lung disease.” (85). During the entire course of Dato-DXd treatment, patients require close monitoring for ILD. Continuous follow-up for ILD until resolution is required regardless of severity. Grade 1 ILD may be managed with systemic corticosteroids (prednisone ≥0.5 mg/kg/day) with potential resumption at 28 days or dose reduction thereafter. Grade 2–4 ILD mandate permanent discontinuation, with hospitalization and high-dose corticosteroids for severe cases. If symptoms do not improve within 3–5 days, additional immunosuppressants may be considered. IRRs occurring within 24 hours post-infusion require rate reduction (Grade 1), temporary interruption with symptomatic treatment (Grade 2), or permanent discontinuation (Grade 3–4). As for the other two types of AESIs, stomatitis and OSTs are predominantly mild to moderate; however, prophylaxis and early detection are of utmost importance (85). Management guidelines recommend a proactive oral care plan initiated prior to first dose, including use of s steroid-containing mouthwash, oral hygiene, and education about the importance of oral hygiene. A physical examination and patient-reported symptoms are sufficient for diagnosis of stomatitis (86). Unless the patient experiences oral mucositis or oral ulceration, no dietary modifications are indicated. The management of Dato-DXd–associated stomatitis should also be guided by the Common Terminology Criteria for Adverse Events (CTCAE) grading system. Dato-DXd associated OSTs include, but not limited to, dry eye, increased lacrimation, blurred vision, conjunctivitis, and keratitis. OSTs necessitate prophylaxis with artificial tears and avoidance of contact lenses, with temporary interruption for Grade 2–3 and permanent discontinuation for Grade 4. Ophthalmologic assessments should be implemented to ensure an accurate diagnosis, proper event grading, and appropriate treatment. Other common adverse events associated with Dato-DXd, including hematological toxicities, gastrointestinal toxicities, and alopecia, occur at lower rates compared to chemotherapy. These events follow conventional CTCAE-guided management protocols. Physicians are required to strictly comply with these operational guidelines, especially regarding preventive measures and monitoring protocols. Such compliance is essential to ensure safe administration of Dato-DXd therapy for patients, enable systematic accumulation of clinical experience, and support the advancement of future clinical trial initiatives.

Several limitations should be acknowledged in this review. First, this manuscript focuses on Dato-DXd, a novel TROP2-targeting ADC. Some data cited herein are derived from conference abstracts, as full publications remain unavailable. Consequently, before complete experimental results are disclosed, the data presented may lack full comprehensiveness. Second, language bias was inherent in our search strategy, as only English-language publications were included. This limitation means that studies published in other languages may have been omitted from our analysis. Future research incorporating non-English sources would help mitigate this constraint.

## 7 Conclusions

Currently, TROP2 ADCs have ushered in a new era of cancer treatment. Dato-DXd has already transformed the treatment landscape for patients with advanced HR+/HER2- breast cancer and those with EGFR-mutated NSCLC in later-line settings. Meanwhile, Dato-DXd monotherapy and combination regimens have demonstrated significant efficacy across diverse clinical settings, including first-line and perioperative treatments for breast cancer and lung cancer. Early-phase clinical studies in other solid tumor types have also yielded promising results. However, these preliminary findings require confirmation through larger, prospective randomized trials before definitive conclusions can be drawn. Given this accumulating evidence, Dato-DXd is poised to potentially bring about substantial changes in the treatment landscape for solid tumors in the near future. Consequently, rigorous toxicity management is paramount, with particular attention required for interstitial lung disease, stomatitis, and ocular adverse events. Healthcare providers should prioritize prophylactic measures and close monitoring. Furthermore, as new TROP2-targeting ADCs continue to be developed, determining the optimal treatment sequencing of TROP2-targeting ADCs including Dato-DXd represents a pressing clinical challenge awaiting relevant trial data. Finally, further research is urgently needed to identify molecular biomarkers predictive of therapeutic response to Dato-DXd and to elucidate the mechanisms underlying primary and acquired resistance, which will guide personalized treatment strategies.
